# Preliminary experience with ketone-targeted treatment of diabetic ketoacidosis

**DOI:** 10.1186/cc10771

**Published:** 2012-03-20

**Authors:** F Riccio, T Drake, S Mathieu, J Cranshaw

**Affiliations:** 1 Royal Bournemouth Hospital, Bournemouth, UK

## Introduction

In May 2011 the Joint British Diabetes Societies (JBDS) published new guidance for managing adult diabetic ketoacidosis. We developed a JBDS-based protocol that measured and treated capillary ketonaemia (not blood glucose) hourly with i.v. 0.1 IU insulin/kg/hour increased by 1 IU/hour if the ketone reduction was <0.5 mM/hour. The final target was capillary ketonaemia <0.3 mM. To allow this insulin rate and avoid hypoglycaemia, 125 ml/hour of 10 or 20% dextrose was started when blood glucose was <14 mM (250 mg/dl). As the effects of this new protocol were unknown, all patients were managed in our high-dependency unit (HDU). We report our experience of the new protocol compared to our old 'sliding scale' insulin titration to blood glucose protocol.

## Methods

We prospectively gathered results of the new protocol over 3 months and performed a chart review of the same results from patients admitted in the previous year managed on wards and the HDU. Results are expressed as median (range).

## Results

Patients on the new protocol (*n *= 7) cleared ketones to <0.3 mM in 8 (7 to 20) hours. The insulin rate needed was 10 (6 to 17) IU/hour. Potassium during treatment was 4 (3.2 to 5.2) mM and required 35 (12 to 60) mM/hour to maintain the target 3.5 to 5 mM. No episodes of blood glucose <4 mM were recorded. Time to reach glucose <14 mM was 7 (1 to 15) hours with a fall rate of 3.7 (2.9 to 6.8) mM/hour. Patients on the old protocol (*n *= 39) were treated for 15 (5 to 20) hours with 3 (0.5 to 6) IU/hour. Potassium during treatment was 3.5 (2.8 to 5.5) mM and required 9 (6 to 16) mM/hour to maintain the target 3.5 to 5 mM. Time to reach glucose <14 mM was 3.5 (1 to 13) hours with a fall rate of 4.2 (0.3 to 13) mM/hour. A total 0.02 results per hour <4 mM were recorded in the old protocol.

## Conclusion

The median insulin infusion rate in an individual patient and the range required to suppress capillary ketonaemia in all patients with diabetic ketoacidosis using this protocol was more than three times that in the old protocol and the amount of i.v. potassium required to maintain near-normal blood potassium during treatment was four times more. There was a slower correction of initial blood glucose. Blood glucose and potassium maintenance during treatment with this protocol would appear to require high-intensity nursing care to maintain patient safety.
